# An easy approach to the Robins-Breslow-Greenland variance estimator

**DOI:** 10.1186/1742-5573-2-9

**Published:** 2005-09-26

**Authors:** Paul Silcocks

**Affiliations:** 1 Trent Research & Development Support Unit, Medical School, Queen's Medical Centre, Nottingham, NG7 2UH UK

**Keywords:** Odds ratio, Variance, Robins-Breslow-Greenland, RBG estimate

## Abstract

The Mantel-Haenszel estimate for the odds ratio (and its logarithm) in stratified case control studies lacked a generally acceptable variance estimate for many years. The Robins-Breslow-Greenland estimate has met this need, but standard textbooks still do not provide an explanation of how it is derived. This article provides an accessible derivation which demonstrates the link between the Robins-Breslow-Greenland estimate and the familiar Woolf estimate for the variance of the log odds ratio, and which could easily be included in Masters level courses in epidemiology. The relationships to the unconditional and conditional maximum likelihood estimates are also reviewed.

## Introduction

The Mantel-Haenszel (MH) estimate for the summary odds ratio across several 2 × 2 tables, *ψ*_*MH*_, was proposed in 1959 [1]. Over twenty years later the lack of a robust estimate for its variance was still being noted [2], yet only a few years afterwards, Robins, Breslow and Greenland introduced their now generally-accepted variance estimator [3] for the Mantel-Haenszel log-odds ratio (denoted by the RBG estimate). This replaced estimation of confidence limits based on the unsatisfactory test-based procedure of Miettinen or the computationally intensive Cornfield type limits which had hitherto been used.

While a useful review of Mantel-Haenszel methods has been published, including some aspects of the historical development towards the RBG estimator [4] the formal derivations by Robins, Breslow & Greenland [3] and Phillips & Holland [5] are not, in the view of this author, easily comprehended. The former omits steps in the argument, while the latter appeals to descending factorial powers. Possibly it is no surprise that even modern textbooks [6,7] merely state the RBG formula without deriving it.

While other variance estimators exist, some are ad hoc, such as the application of the cohort study formula to case-control data suggested by Clayton and Hills [8], only apply to the large few strata case [9] or are closely related to the RBG estimator [10]. One rather different exception is Sato's formula [11] but this procedure gives confidence limits directly in the odds ratio scale.

It is the intention of this article to present an informal derivation of the RBG estimator as an extension of the familiar variance formula of Woolf [12], and which could readily be included in standard textbooks of epidemiology or biostatistics. I will describe this from the perspective of a case-control study.

## Analysis

### How does the Mantel-Haenszel estimate arise?

Consider a stratified case-control study for which the i^th ^of k independent tables is:



Neglecting constants, the unconditional likelihood for the i^th ^table is:



where in the i^th ^table *θ*_*i *_= probability of exposure if a case and *ø*_*i *_= probability of exposure if a control.

The maximum likelihood estimate (MLE) for *ø*_*i *_is given by *b*_*i*_/(*b*_*i *_+ *d*_*i*_) and if we re-parameterise *θ*_*i *_as *ψø*_*i*_*/ *[*ψø*_*i *_+ (1 - *ø*_*i*_)], where *ψ *is the odds ratio (assumed common to all tables), the contribution to the overall log likelihood made by terms involving *ψ *is:

∑ *a*_*i *_ln {*ψø*_*i*_/[*ψø*_*i *_+ (1 - *ø*_*i*_)]} + *c*_*i *_ln {1/[*ψø*_*i *_+ (1 - *ø*_*i*_)]}.

Differentiating with respect to *ψ *and equating to zero, and rearranging (noting that *a*_*i *_+ *c*_*i *_= *n*_1*i*_)

we obtain:

∑ *a*_*i *_ln {*ψø*_*i*_/[*ψø*_*i *_+ (1 - *ø*_*i*_)]} + *c*_*i *_ln {1/[*ψø*_*i *_+ (1 - *ø*_*i*_)]}.

i.e., ∑ {*a*_*i *_- *n*_1*i*_*ψø*_*i*_/[*ψø*_*i *_+ (1 - *ø*_*i*_)]} = 0

i.e., ∑ {[*ψa*_*i*_*ø*_*i *_+ *a*_*i *_- *a*_*i*_*ø*_*i *_- *n*_1*i*_*ψø*_*i*_]/[*ψø*_*i *_+ (1 - *ø*_*i*_)]} = 0.

This must be solved numerically to obtain the MLE for *ψ*, but if the denominators do not vary too much across the tables we merely have to solve:

∑ [*ψa*_*i*_*ø*_*i *_+ *a*_*i *_- *a*_*i*_*ø*_*i *_- *n*_1*i*_*ψø*_*i*_] = 0

i.e., ∑ [*ψ*(*a*_*i *_- *n*_1*i*_)*ø*_*i *_+ *a*_*i *_(1 - *ø*_*i*_)] = 0

or, ∑ *a*_*i *_(1 - *ø*_*i*_) = ∑ *ψ*(*n*_1*i *_- *a*_*i*_)*ø*_*i*_

giving, ∑ *a*_*i *_(1 - *ø*_*i*_) = *ψ *∑ (*n*_1*i *_- *a*_*i*_)*ø*_*i*_

and since, *ψ*_*i *_= *b*_*i*_/(*b*_*i *_+ *d*_*i*_) = *b*_*i*_/*n*_0*i*_



This can be used as a first approximation to find the MLE (if there is only one table then *ψ is *the unconditional MLE = ad/bc). Now in stratified case-control studies with a constant ratio, *r*, of controls to cases, the total number of subjects in each stratum is given by *n*_*i *_= *n*_0*i *_(1 + *r*), so *n*_0*i *_= *n*_*i*_/(1 + *r*). A constant *r *will be achieved by design if there is caliper matching; otherwise – as with a post-stratified analysis – this will be only approximately true. The term (1 + *r*) can then be cancelled and we are left with:



The MH estimator is therefore a first approximation to the unconditional MLE in the large strata case with a constant control:case ratio across strata. However the MH estimator actually *coincides *with the *conditional *MLE for the matched pairs design, as outlined, for example on page 164 of Breslow & Day [2].

The sensitivity to variation in the *ø*_*i *_and constancy of the control:case ratio is not high, as shown by the data in Table 1. In a sense this would be expected because for the most sparse (e.g., pair-matched) data the control:case ratio will be constant, and while the *ø*_*i *_then have maximum variance – being only 0 and 1, the MH estimate coincides with the conditional MLE. Conversely, for large strata the control:case ratio will vary, but the variance of the *ø*_*i *_will be less and the MH estimate will then approximate the unconditional MLE.

**Table 1:** Simulated case-control data with true odds ratio = 5

Case	Control	*ø*	Controls:cases
36	97	0.58	4
6	71		
42	168		
			
Ca	Co		
41	79	0.94	2
1	5		
42	84		
			
Ca	Co		
2	1	0.02	2
26	55		
28	56		
			
Ca	Co		
19	25	0.30	3
9	59		
28	84		
			
Ca	Co		
20	41	0.37	4
8	71		
28	112		
			
Ca	Co		
30	21	0.62	1
4	13		
34	34		
			
Ca	Co		
22	26	0.46	2
6	30		
28	56		

Odds ratio estimates (Stata v7.0):

Mantel-Haenszel 4.38 (95% CI 2.85 to 6.72)

Conditional MLE 4.36 (95% CI 2.85 to 6.67)

Unconditional MLE 4.42 (95% CI 2.88 to 6.78)

### Deriving the variance of the Mantel-Haenszel estimate

Consider again the i^th ^2 × 2 table, giving the frequencies in each cell:



For odds ratio , estimated for a single table by the cross-product ratio *a*_*i*_*d*_*i*_/*b*_*i*_*c*_*i*_, application of the delta method gives Woolf's logit-based formula [8]:

 with *n*_*i *_= *a*_*i *_+ *b*_*i *_+ *c*_*i *_+ *d*_*i *_and, 

The delta method is a widely used procedure in statistics when an approximation is needed for the variance of a function of a variable whose variance is known. In this instance the variable with known variance is a proportion *p*, and the function is the logit. The basic delta method formula is: var(*y*) ≈ (*dy*/*dx*)^2 ^var(*x*) from which, if *y *= *logit*(*p *= ln[*p*/(1 - *p*)],

var(*y*) ≈ (1/*p *+ 1/(1 - *p*))^2 ^*p*(1 - *p*)/*n*

= (1/*p *+ 1/(1 - *p*))1/*n*

= (1/*a *+ 1/*b*)

if *p *= *a*/*n *and *n *= *a *+ *b*.

Here we have two independent proportions (the proportion of cases and controls exposed) and Woolf's formula is obtained by estimating the variances of the separate logits and adding them.

For k such 2 × 2 tables, each representing a separate stratum, the Mantel-Haenszel pooled estimate of the *common *odds ratio *ψ *is given by:



Hence *ψ*_*MH *_is a weighted average of the stratum-specific odds ratios. The weights approximate the inverse of the variance of each _*i *_if the true value of *ψ *= 1. Note that the assumption here of a common odds ratio is not required for the Mantel-Haenszel *test*.

To derive the variance, in addition to the approximation involved in application of the delta rule, an assumption is also made that each stratum-specific odds ratio is close enough to the Mantel-Haenszel pooled estimate to permit terms like *a*_*i*_*d*_*i*_/*b*_*i*_*c*_*i *_to be replaced by *ψ*_*MH*_.

We then proceed by obtaining an approximation which avoids zeros in the formula for var[ln()]. The motivation for this can be seen by comparing the weights for *ψ*_*MH *_– which are unaffected by zeros except for deleting such strata – whereas if Woolf's variances were used, the result would be indeterminate if cells with zeros were present.

Taking the weights as constant,



Assuming a common odds ratio *ψ*, estimated by *ψ*_*MH*_, this can be written as:



Leading to a formula suggested by Hauck [9]:



As mentioned above, a problem with this formula is that it fails if cell entries are zero. However we can proceed further by re-writing the formula as:



On substituting 1/*ψ*_*MH *_for (*b*_*i*_*c*_*i*_/*a*_*i*_*d*_*i*_):



Now if the rows of the 2 × 2 table are interchanged, the variance stays the same. But a similar argument to that above leads to:



(Note that the new odds ratio  formed by exchanging rows is just 1/*ψ*_*MH*_.) "The" variance, V, of ln(*ψ*_*MH*_) is therefore taken to be the mean of the two estimates [13] as follows:

Let R = ∑ (*a*_*i*_*d*_*i*_/*n*_*i*_) and S = ∑ (*b*_*i*_*c*_*i*_/*n*_*i*_). On substituting into the two variance formulae:



Next, divide the top and bottom by S^2 ^and move the  term outside the brackets to obtain:

 which is eq. 9 in Phillips & Holland [5].

If we now put

P_i _= (*a*_*i *_+ *d*_*i*_)/*n*_*i *_and Q_i _= (*b*_*i *_+ *c*_*i*_)/*n*_*i *_with R_i _= *a*_*i*_*d*_*i*_/*n*_*i *_and S_i _= *b*_*i*_*c*_*i*_/*n*_*i*_

then 

which on multiplying out the brackets, rearranging and noting that R/S = *ψ*_*MH*_, gives:



This is the RBG formula!

When there is only one stratum, this reduces to (1/*a *+ 1/*b *+ 1/*c *+ 1/*d*) which is the familiar logit based formula of Woolf and which approaches 0 as the sample size increases, assuming a finite true odds ratio. Clearly as the RBG variance estimate is a finite sum of such estimators the RBG estimate will also approach 0, for large strata.

The RBG estimator was derived above on the assumption that the stratum-specific odds ratio estimates could be liberally replaced by the common value, in turn estimated by *ψ*_*MH*_; both assumptions are reasonable with large samples per stratum. However, the success of the RBG formula derives from its being applicable also to the sparse data case.

To see this, consider a matched-pairs case control study. The capital letters denote the frequency of case-control pairs.



In such a study each stratum has only two observations. The table can be decomposed into four types of "unmatched" table according to the exposure category of the case and the control, the frequency of each type being given by the frequency of the corresponding case-control pairs:





Only the B such tables with *a*_*i *_= *d*_*i *_= 1 and the C such tables with *b*_*i *_= *c*_*i *_= 1 contribute to the estimate of the odds ratio. Note that these are disjoint sets of tables.

Under these circumstances: *ψ*_*MH *_= B/C which coincides with the conditional MLE and:

a) the middle term of the RBG formula vanishes because if *b*_*i*_*c*_*i *_= 1 then (*a*_*i *_+ *d*_*i*_) = 0, and if *a*_*i*_*d*_*i*_*= *1 then (*b*_*i*_*+ c*_*i*_) = 0

b) R = ∑ *a*_*i*_*d*_*i*_/*n*_*i *_*= *B/2 & S = ∑ *b*_*i*_*c*_*i*_/*n*_*i *_= C/2

c) There are B terms in which *a*_*i*_*d*_*i *_(*a*_*i *_+ *d*_*i*_) = 2 C terms in which *b*_*i*_*c*_*i *_(*b*_*i *_+ *c*_*i*_) = 2

giving:

V = B/B^2 ^+ C/C^2 ^= 1/B + 1/C

This is not only the familiar logit based formula for the variance of the log odds ratio for matched pairs, but is also the variance of the conditional maximum likelihood estimate. This is asymptotically consistent from the general properties of a MLE (and it's easy to see that as the number of tables increases, V → 0).

In other words, the RBG formula, though derived here without assuming validity in the sparse case, does in fact possess this property.

Table 1 shows how closely the conditional maximum likelihood estimate, unconditional maximum likelihood estimate, and MH estimate agree, despite varying *ø*_*i *_and control:case ratio.

## Conclusion

The Mantel-Haenszel estimate of the odds ratio approximates the maximum likelihood estimate for large, few strata and coincides with the conditional maximum likelihood estimate for the sparse data (matched pairs) case.

The RBG formula is the estimator of choice for the variance of the Mantel-Haenszel log-odds-ratio because it applies both in the large few strata case and in the many sparse strata case (as in matched pairs analysis), when the RBG variance estimate actually coincides with the conditional maximum likelihood variance estimate.

Moreover the RBG formula reduces to familiar standard forms for a single stratum and for matched pairs.

Formal derivation of the RBG formula is tricky but an informal, accessible derivation is possible as outlined above, which uses nothing more advanced than the delta method for approximating a variance.

## Competing interests

The author(s) declare that they have no competing interests.
